# Comparing legislation for involuntary admission and treatment of mental illness in four South Asian countries

**DOI:** 10.1186/s13033-019-0322-7

**Published:** 2019-10-24

**Authors:** Sangeeta Dey, Graham Mellsop, Kate Diesfeld, Vajira Dharmawardene, Susitha Mendis, Sreemanti Chaudhuri, Aniruddha Deb, Nafisa Huq, Helal Uddin Ahmed, Mohammad Shuaib, Faisal Rashid Khan

**Affiliations:** 1 Waikato Hospital, Waikato District Health Board, 222 Pembroke Street, Hamilton, 3240 New Zealand; 2 Waikato Clinical Campus Waikato DHB, Auckland University, Auckland, New Zealand; 3 Auckland University of Technology, Auckland, New Zealand; 4 District General Hospital, Matara, Sri Lanka; 5 Teaching Hospital, Galle, Sri Lanka; 6 Psychiatric Nursing Home Trust, Kolkata, West Bengal India; 7 School of Public Health, Independent University, Dhaka, Bangladesh; 8 Department of Child, Adolescent and Family Psychiatry, National Institute of Mental Health, Agargaon, Dhaka, Bangladesh; 9 Waikato Hospital, Hamilton, New Zealand; 10 Al Nafees Medical College, ISRA University, Islamabad, Pakistan

**Keywords:** Mental health legislation, Law, South Asia, Comparison, Trend

## Abstract

**Background:**

Involuntary admission or treatment for the management of mental illness is a relatively common practice worldwide. Enabling legislation exists in most developed and high-income countries. A few of these countries have attempted to align their legislation with the United Nations Convention on the Rights of Persons with Disabilities. This review examined legislation and associated issues from four diverse South Asian countries (Bangladesh, India, Pakistan and Sri Lanka) that all have a British colonial past and initially adopted the *Lunacy Act* of 1845.

**Method:**

A questionnaire based on two previous studies and the World Health Organization checklist for mental health legislation was developed requesting information on the criteria and process for involuntary detention of patients with mental illness for assessment and treatment. The questionnaire was completed by psychiatrists (key informants) from each of the four countries. The questionnaire also sought participants’ comments or concerns regarding the legislation or related issues.

**Results:**

The results showed that relevant legislation has evolved differently in each of the four countries. Each country has faced challenges when reforming or implementing their mental health laws. Barriers included legal safeguards, human rights protections, funding, resources, absence of a robust wider health system, political support and sub-optimal mental health literacy.

**Conclusion:**

Clinicians in these countries face dilemmas that are less frequently encountered by their counterparts in relatively more advantaged countries. These dilemmas require attention when implementing and reforming mental health legislation in South Asia.

## Background

Many countries have mental health legislation (MHL) that can authorize involuntary mental health assessment and/or treatment. The World Health Organization (WHO) regards such legislation as a key component of good health governance [1].

The basis of modern mental health law originates from English statutes from the reign of Edward 1st in the late thirteenth century [2]. The entwinement of the doctrine of ‘parens patriae’ and the ‘police powers’ of the state were important features of early mental health laws. Parens patriae translates as ‘parent of the country’, justified detaining and/or treating a person compulsorily on the basis that the person was not able to look after their own interests [2]. The ‘police powers’ justified intervention as protecting other people from the person deemed ‘mad’, typically from physical violence [2]. In modern legislation, ‘risk of harm to self or others’ remains the basis of involuntary admission and treatment.

Since the late 1970s, MHL has become increasingly influenced by international human rights law [3]. In 1991, with the adoption of the Principles for the Protection of Persons with Mental Illness (MI Principles), the journey of ensuring least restrictive care began [3]. The United Nations Convention on the Rights of Persons with Disabilities (UN-CRPD) adopted in 2006 [4] is a potent platform for protection and has been ratified by 177 countries thus far.

However, many developing countries, alongside some developed countries, have not yet reformed or updated their MHL to align with international human rights conventions. In addition, bodies to regulate or monitor mental health laws exist in only a few countries. For example, the WHO found that more than 65% of countries in low and lower middle-income groups did not have an independent monitoring body [1].

In the past 5 years, several countries in the South Asian region undertook legal reform. This was partly in response to the WHO’s comprehensive mental health action plans and the global mental health movement [5]. The four countries (Bangladesh, India, Pakistan and Sri Lanka) included in this review belong to the South Asian region and are considered developing countries according to the WHO. All of these countries have had phases of British colonial rule and inherited the 19th century British *Lunacy Act*. In 1947, after the division of British India into Pakistan and India, both countries adopted the 1912 version of the *Lunacy Act*, and Bangladesh adopted the *Lunacy Act* when it became independent in 1971. In Sri Lanka, the Act was named the *Ceylon Lunacy Ordinance* in 1873 [6].

However, all of these countries have travelled a long way politically, socially and economically. The *Lunacy Act*, like any old legislation, is not informed by modern day human rights law or psychiatric practice. The Act is described as ‘archaic and obsolete’ [7].

This article compares the MHL and some relevant concerns regarding involuntary assessment and treatment for people suffering with mental illness in these four countries. While their colonial heritages are similar, their healthcare systems vary considerably [8–10]. Despite this variation in healthcare systems, their goals to develop mental health services and reform their legislation in order to ensure proper care for this vulnerable group are comparable. Notwithstanding geographical, cultural, historical and linguistic diversity, commonalties have been identified when laws and some psychiatric clinical practices have been compared across nations [11–14].

## Method

Based on two previous studies and the WHO checklist for MHL [12, 13, 15], standardized questions requesting information regarding the law that governed involuntary assessment and treatment were developed (see Additional file 1: Appendix S1).

The four key coordinating participants for each country were identified through the lead author’s professional network. Three of these participants were psychiatrists (from India, Pakistan and Sri Lanka) and one (from Bangladesh) participant was a public health professional with special interest in the area. These participants then identified other local collaborators who were selected for their expertise either due to their special interest in this area or due to their eagerness to participate in this project.

Altogether there were nine participants (Three from India, two from Bangladesh, two from Pakistan and two from Sri Lanka). One participant from India requested to remain anonymous. A letter with participant information and questionnaire was sent to the four key coordinating participants.

The completed questionnaire was returned to the lead author who reviewed the relevant MHL in comparison to the participants’ responses. The coordinating participant from each country was responsible for reviewing the findings and ensured all other participants were in an agreement. The participants from each country also had opportunity to comment about their concerns regarding the legislation or associated issues.

## Results

The findings for each country are summarized below.

## Summary of legislation for involuntary admission and treatment process

### Bangladesh

After 106 years Bangladesh replaced the *Lunacy Act* 1912 with *The Mental Health Act Bangladesh 2018* [16]. This new Act defines the criteria for involuntary admission and removed terms such as ‘lunatics’ and ‘temporary patients.’ The criteria are based on severity of illness, refer to risk to themselves or others and includes poor self-care and noncompliance of treatment. Mental illnesses associated with substance abuse disorders or intellectual disabilities are included as criteria for detention.

A relative, parent or friend can initiate an application for involuntary admission. This is followed by an assessment by a medical officer within 24 h. A medical officer can authorize emergency admission for up to 72 h. An assessment by a psychiatrist is required for ongoing involuntary admission. This status is reviewed every 28 days. The maximum duration of admission is 180 days. After this, a Mental Health Review and Monitoring Committee can extend the duration of stay if necessary.

There is intention to establish Mental Health Review and Monitoring Committees in every district. These committees will include government representatives and mental health clinicians. Relatives and parents of patients may appeal to this committee if they are not satisfied with treatment. Both private and government hospitals must be licensed for admitting and treating involuntary patients. The government funds legal representation for the patient. A medical practitioner will face disciplinary action if it is found that a false certificate for mental illness has been provided. There is no community extension of this legislation.

The current law is very new to clinicians in Bangladesh, and participants from Bangladesh have not expressed any specific concerns about the law itself and did not comment on implementation issues specific to Bangladesh. The new law has rather created hope among clinicians and especially the law’s reference to ‘rights of patient’ is regarded as encouraging. English translation of the current law is currently unavailable.

### India

In 1950, 3 years after independence, the Indian Psychiatric Society first submitted a revision of the *Lunacy Act* 1912. After protracted debate, the *Mental Health Act* 1987 came into operation in 1993. Most recently, the *Mental HealthCare Act* 2017 (*MHA* 2017) [17] has been enacted.

Involuntary admission was replaced by supported admission in the *MHA* 2017, providing for appointment of a representative nominated by the patient for supported decision-making. At any time, the patient may revoke this appointment.

The supported admission requires two assessments; one by a psychiatrist and one by a mental health professional or medical practitioner. Both assessors are required to examine the person independently on the day of admission or in the preceding 7 days. There is also scope for emergency hospitalization for 72 h which can be authorized by a registered medical practitioner until the person has been assessed by a mental health professional.

For monitoring, the Mental Health Review Board (MHRB) must be informed within 7 days of a supported admission, and the person, their representative, or an appropriate organization may appeal this decision.

No formal review is required before 30 days. If continued hospitalization is required after 30 days, the MHRB undertakes a review of whether this is justified. The State Mental Health Authority (SMHA) and the Central Mental Health Authority (CMHA) confer with the MHRB when required. The CMHA maintains a register of all mental health establishments, develops quality and service standards for the establishments, and trains all persons regarding the provisions and implementation of the Act.

The Act provides guidance to ensure informed consent of the patient with the support of their nominated representative. In this case, mental health professionals are required to review the capacity of the person to give consent every 7 days. Advanced directives are allowed to cover future situations where the patient may cease to have capacity. This supported admission is a shift from substituted decision-making. There is no community extension of this legislation.

The contributors from India expressed several concerns about the legislation:

- The ‘right to refuse treatment’ would be unlikely to be accepted either by the patient’s family or mental health professionals because the concept of personal independence is reported as different culturally; ‘family preferences often supersede the personal.’ This may affect the management of any unwilling patient that requires treatment.
- Reality on the ground (implementation of the Act) was reported as being different despite police and judiciary services receiving training.
- Concerns were raised about absence of a clear definition of personality disorder and substance abuse, absence of clear safeguards for emergency situations, lack of clarity around review processes, absence of community extension, or any support mechanism to enable people to make informed decisions.
- The legislation requires hospitals dealing with mentally ill people to be licensed and this may make it very difficult for general hospitals to cater for the mentally ill. Due to stigma, many patients may not want to be admitted to a specialized mental health care facility or a patient in general hospital may develop a mental health issue and may not receive treatment due to absence of a license. According to participants this may cause more confusion.
- Last but not the least is concern about local funding as both central and state governments have responsibilities. According to one participant, ‘focus is on the content of the legislation rather than its effect. Implementation is a key issue.’ For example, 28 years after enactment, only 11% of Indian states had state mental health rules. Specific measures need to be in place to address funding, staffing, public health priorities and stigma.

### Pakistan

Pakistan adopted the *Lunacy Act* 1912 from British India when they became independent in 1947. It was replaced by *The Mental Health Ordinance* 2001 (MHO 2001) [18]. This Act set out to: ‘amend the law relating to the treatment and care of mentally disordered persons, to make better provision for their care, treatment, management of properties and affairs and to encourage community care and further to provide for promotion of mental health and prevention of mental disorder’ [18].

The MHO 2001 dealt with access to mental health care, voluntary and involuntary treatment, competency, capacity and guardianship issues. The ordinance also addressed human rights issues and informed consent. Under this ordinance, a Federal Mental Health Authority (FMHA) was founded in 2001 to develop national standards of care.

Mental disorder in this ordinance means mental illness, severe personality disorder, and severe mental impairment. There are four types of detention of a patient according to the MHO 2001, namely:

- admission for assessment (28 days).
- admission for treatment (6 months).
- urgent admission (72 h).
- emergency holding (24 h).

The ordinance allows a patient’s relatives/family members to appeal against the order of detention to a court of protection within a period of 14 days.

The MHO 2001 requires assessment by a psychiatrist (or a medical practitioner with experience in psychiatry) and a medical practitioner for involuntary admission and treatment. ‘Emergency powers’ allow a clinician to provide treatment without invoking the legislation.

A government-established board is required to periodically inspect every part of the psychiatric facility and examine as far as possible every patient and mentally disordered patient. The board may make recommendations to a psychiatric facility, the provincial mental health authority, or the government regarding conditions in the facilities. The Board of Visitors consists of a chairperson (a judge of the High Court), two psychiatrists (one with 10 years’ minimum experience, one prominent citizen of good standing), two medical practitioners (with a minimum standing of 12 years), and the Director of General Health Services (or the Director’s nominee).

However, health is now governed at a provincial level and the FMHA was dissolved in 2010. The ordinance was replaced by the Mental Health Act. Sindh province of Pakistan enacted the law in 2013, followed by Punjab in 2014 and Khyber Pakhtunkhwa in 2017.

*The Sindh Mental Health Act* 2013 is based on the MHO 2001. In this legislation, mental disorder ‘means a mentally ill person who is in need of treatment by reason of any disorder of the mind other than mental impairment and severe personality disorder’ [19]. The types of detention are similar to those outlines in the MHO 2001.

For monitoring, the Sindh Mental Health Authority consists of a chairperson and not more than fourteen government appointed members. It is required to advise government on all matters relating to mental health including the prescribing code of practice for achieving the purposes and objects of the Act. The Sindh Mental Health Authority in consultation with government establishes the Board of Visitors (as per MHO 2001) for carrying out the purposes of the Act. This Act has addressed the assessment and treatment of a ‘mentally disordered’ accused person detained in prison but does not include those under blasphemy laws (laws prohibiting speaking insultingly about a religion or god).

There is also a *Punjab Mental Health Act* 2014 [20] which is an amendment of the MHO 2001. The Punjab Mental Health Authority replaced the FMHA. The authority consists of a chairperson and not more than 10 members appointed by the Punjab Government. Assessment and treatment process are similar to those in the MHO 2001. The Khyber Pakhtunkhwa Mental Health Act 2017 is also similar and based on MHO 2001.

There are no community extensions of the *Sindh*, Punjab *and Khyber Pakhtunkhwa Mental Health Acts*, but the Acts do refer to ‘provision of guidance, education, rehabilitation after care and preventative measures in the community.’

Participants from Pakistan raised concerns that those held in custody under blasphemy laws did not have any rights in this legislation. This is now included as ‘A person who attempts suicide including an accused of blasphemy shall be assessed by an approved psychiatrist and if found to be suffering from a mental disorder shall be treated appropriately under the provisions of this Act.’ (Chapter VII, clause 49). Apart from general concerns about implementation, no other specific concerns were raised by the two participants.

### Sri Lanka

The current legislation is the *Mental Diseases Ordinance* 1956 [21] first enacted in 1873. This is based on the *Lunacy Ordinance* of 1873 and mainly regulates the custody, hospitalization and detention of people with mental illness. This remains an ordinance (an ordinance is mostly referred to as local level laws that have the same power and effect as that of acts, although only at a local level) and has not been replaced by a legislated mental health act.

This law is still operating with minor modifications. There are two categories for detention. First, the presence of an unsound mind defined as: ‘Every person shall be deemed to be of unsound mind who is so far deranged in mind as to render it necessary that he, either for his own sake or that of the public, should be placed under control^’^.

The assessment of an unsound mind is undertaken by a civil court enquiry and is open to judicial appeal. A certificate by a medical practitioner should accompany an application by a person to the district court. The court continues the inquiry and hears the evidence. It may then either discharge or remand the person in custody or in a mental asylum for further observation. If any fit family member or friend is prepared to take responsibility for the person of unsound mind, the court can order that the person be released to the relative.

Second, there is the concept of a temporary patient: ‘A person who is suffering from mental illness and is likely to benefit by temporary treatment in a mental hospital but is for the time being incapable of expressing himself as willing or unwilling to receive such treatment may be received under this section as a temporary patient for the purpose of treatment’.

A court is not involved in this process. The spouse, relative or any other person can submit an application to the superintendent of the hospital accompanied by recommendations from two medical practitioners (with no greater interval than 5 days between examining the person and submitting the application). The order expires 14 days after the date when the last medical practitioner examined the person. The person may be committed for up to 1 year. If the temporary patient becomes capable of expressing themselves, then they shall not be detained for more than 28 days unless circumstances change.

Although the legislation does not specify that the assessor must be a psychiatrist, in practice, a psychiatrist (or medical practitioner working under a psychiatrist) is usually involved in the decision-making. District Court admissions for patients of unsound mind are, in current practice, mostly reserved for persons with mental illness who are homeless, found wandering and not safe.

The legislation is silent regarding human rights. However, the Mental Health Policy of Sri Lanka 2005 has a rights-based approach [22]. The policy calls for new legislation to incorporate human rights for the detained person.

Participants raised concerns about the fact that this legislation is outdated and roles of clinicians (including psychiatrists) are not clearly defined. There is also no provision for automatic independent review. Participants however reported that mental health literacy has improved in Sri Lanka, but due to bureaucratic processes and lack of consensus among stakeholders, several attempts to develop a new mental health act have been aborted. The draft MHL in 2007 incorporated human rights safeguards, eliminated obsolete terminology, and focused on rehabilitation and the capacity to consent.

## Discussion

Comparisons between MHL can be problematic, as each is formed within a particular social, legal, political and economic context. The situation and challenges are significantly different in developing countries compared to developed countries. Most literature relating to MHL is in the context of economically advantaged countries, ‘where modern legal forms flow from a broadly post-enlightenment mentality, where individual rights and liberties are the stuff of national identity’ [23]. This is not the reality for many developing countries and legislation needs to be interpreted within their current sociopolitical and cultural contexts.

The review of the MHL and associated issues in the four countries revealed both similarities and differences. Despite their historical and cultural differences, they all started with adoption of the earlier British *Lunacy Act*. There is also much in common in terms of where they have got to or where they are trying to go. However, the rates at which they have moved closer towards UN and WHO principles or recommendations vary considerably. Yet they share common aims and, in some respects, achievements. All but Sri Lanka have reformed their legislation.

The *Bangladesh Mental Health Act* 2018 replaced the *Lunacy Act* 1912. This is a major milestone for Bangladesh. This development came not long after India enacted their *Mental Health Care Act* in 2017. Pakistan also replaced the MHO 2001 with provincial *MHA*s in the last 5 years. Therefore, most of these developments occurred after introduction of the UN-CRPD. As a result, all of these countries have tried to develop legislation with a degree of alignment with international human rights.

The definitions and criteria for involuntary detention or supported admission (as per Indian *MHA* 2017) are clearer in all three legislations. Pakistan is the most specific about assessment, treatment, as well as emergency detention compared with Bangladesh and India. In all three laws the criteria are similar, based on risk and presence of mental illness/disorder and where treatment is indicated. Assessment processes are also very similar. All legislation clearly identifies the role of psychiatrists in the process. Given the insufficient supply of psychiatrists in these countries, the legislation specifies the role of medical officer with special training in psychiatry when a psychiatrist is not available or accessible. However, for prolonged detention, all legislation requires assessment by a psychiatrist.

Including the role of MHRBs or committees as watch dogs is a significant milestone, as this ensures proper utilization of legislation and reduces the chance of abuse of human rights. Opportunities for families/caregivers to appeal against the detention have been addressed within specific timeframes. With regards to informed consent, capacity and advanced directives, the Indian *MHA* 2017 is more specific. Pakistan and Bangladesh have addressed these crucial areas less specifically and with less elaboration in their documents. Countries carry ethical and moral responsibilities to ensure financial support for ongoing treatment and care in the developed world [24]. Even though it is an area of contention due to its implication on countries’ finances and resources, all legislation refers to legal fees and financial support from governments.

The Bangladeshi and Indian legislation are also clear about requiring institutional licenses to treat involuntary patients. This may possibly create ongoing issues due to the complexities of public and private sectors in all of these countries, as well as urban and rural areas. Resource allocation and financial implications of such laws are likely to be debated. However, for the first time an attempt has made in legislation to address both private and public sectors.

New and more progressive approaches are noticeable in India’s *MHA* 2017 compared with the two others. It has replaced terms such as ‘involuntary’ or ‘compulsory’ with the ‘supported admission’ and UN-CRPD is the major catalyst for this Act with rights of persons with disabilities at its core [24]. The inclusion of informed consent, advanced directives and nominated representatives for supported decision-making are changes to address human rights violation and prolonged detention. Theoretically, the Indian *MHA* 2017 is considered as a progressive piece of legislation, concordant with a higher proportion of the WHO human rights standards than the current legislation of England and Wales [15, 25].

Compared with the Indian *MHA* 2017, the human rights of people with mental illness are not adequately addressed in the Bangladeshi legislation despite being the newest [26]. There is no human rights review body in Bangladesh to oversee regular inspections of mental health facilities as noted a decade ago [27]. Pakistani legislation has addressed human rights and informed consent in definition, but not as extensively addressed as in India.

In contrast, despite being new, none of this legislation has managed to develop robust clinical review processes. Review is only required after 28 or 30 days, and sometimes longer. For example, detention in Pakistan for treatment requires a review after 6 months which is much longer than in developed countries. Although community support and rehabilitation are referenced, none of the legislation includes any community extension. This is in contrast to most developed countries despite equivocal evidence of effectiveness for such community treatment orders.

The *Bangladesh Mental Health Act* also states that medical practitioners may be fined if they provide false certificates of mental illness or treat patients in unlicensed institutions. Concerns that this may create fear within an under-resourced, struggling, health system have been noted [28]. In the Indian *MHA* 2017, the shift of responsibility to a nominated representative instead of professionals is seen as not in alignment with a culture that is still driven by ‘collectivist value’ (emphasis on cohesiveness among individuals and prioritization of the group over self). The concern is that this may affect sometimes already strained relationships in families due to illness burden and caregiver stress. Treatment may not occur due to poor mental health literacy [29].

In summary, the three new pieces of legislation in this region have made significant progress. The legislation has begun to incorporate human right issues but despite being in the same region and following the same guidelines, the inclusion of these terms is variable. Psychiatric advanced directives and shared decision-making are seen as two fundamental tools to safeguard the person’s choice, dignity and autonomy [30]. Apart from the Indian *MHA* 2017, none of the other legislations address this adequately.

In contrast, Sri Lanka is still practicing centuries old legislation that has not incorporated anything from modern psychiatry. It is therefore difficult to compare their legislation with the other three countries. However, Sri Lanka has made significant progress in the delivery of mental health care and development of a mental health policy.

The current legislation continues to use terms such as ‘unsound mind’, ‘lunatics’ and the process is confusing due to the inclusion of two types of patient, rather than defining mental illness or disorder. Absence of regular review is also a concern, and similar to the three new pieces of legislation is an absence of any community extension.

More concerning is that although Sri Lanka is a signatory of the UN-CRPD since 2016, its existing legislation does not talk about human rights issues. Their legislation has not been updated since the UN-CRPD has been in place. Signing the document states that the country agrees to align their domestic legislation with the UN-CRPD principles [4]. The necessary steps to implement the UN-CRPD are detailed, such as Article 4.1. ‘(b): To take all appropriate measures, including legislation, to modify or abolish existing laws, regulations, customs and practices that constitute discrimination against persons with disabilities.’

The current legislation of Sri Lanka, derived from Great Britain, does not adequately reflect modern Sri Lanka’s sociopolitical or cultural context. Since the amendment in 1956, Sri Lanka has experienced civil uprisings, ethnic conflict, a devastating tsunami and bombing in 2019. It is argued that given the social, political and economic changes, the current legislation for involuntary admission warrants reform [6]. The current legislation promotes a more custodial approach and institutionalized care, where rights of the individual with mental illnesses have not been addressed. However, the draft MHL2007 has been awaiting approval for more than 10 years due to difficulties in reaching consensus between different interest groups or stakeholders.

Overall, despite the similarities and differences in legislation, all these four countries share some common concerns regarding the practical aspect of implementing their legislation. These need consideration while developing or reforming a new law. For example, poorly developed mental health services, poor mental health literacy and lack of adequate resources. It is worth noting that 28 years after the enactment of the *Mental Health Act* 1987 in India, only 11% of Indian states have state mental health rules in place and possibly many states are unaware of these rules [31]. Therefore, people with mental illness continue to be potentially vulnerable to various type of abuse and violation of their rights. Reform of legislation would need to go hand in hand with resource issues and service improvement [32].

In addition to this, delays in approval or enactment sometimes occur due to lack of agreement amongst all stakeholders. One current example of this is in Sri Lanka. Despite significant development in providing care and developing their mental health policy, particularly due to bureaucratic process, Sri Lanka has been waiting for approval of their draft *Mental Health Act* for more than 10 years and is forced to practice archaic legislation in a modern world.

Finally, it is important to mention that cultural and religious beliefs such as supernatural influences are considered by many people as a cause of mental illness in this region. Rather than professionals, religious healers usually attend patients first [33]. Also, as mentioned by the participants from India, collectivist value in the culture may not be in alignment with the ‘autonomy to refuse treatment’ in this region. Collectivist values that emphasize communality and mutual dependence over the autonomy of the individual dominate decision-making in this region. The collectivist value complicates the management and direct application of some international ethics codes [34]. Therefore, it has been asked ‘would these countries be better served by a different model of reform of MHL compared with developed countries?’ [23]. Even though cultural differences cannot be ignored, it is also important not to use them to mask stigma and oppression [23].

## Conclusion

In the 21st century we are still dealing with stigma of mental illness in both the developing and developed world [35]. Challenges in daily practice are different in the South Asian region from those in the developed countries.

This review highlighted many concerns common to the four countries. It is clear that account needs to be taken of the context and everyday realities before drafting and formalizing MHL. The countries included in this paper are slowly but surely addressing their MHL in the lights of concerns about custodial philosophy and human rights violation. The findings suggest three countries have reformed their legislation following WHO guidance and have also incorporated human rights issues. However despite their common legal heritage, how they are reforming their laws are influenced by their individual socio-political scenario. The criteria and processes for involuntary admission in all three new legislations are similar. They also have acknowledged shortages of specialists, resources in rural areas as well as private and public sectors. However, concerns remain the same due to probable failure to invest politically and financially. This could lead to further unsuccessful attempts to improve care for these vulnerable groups. Sri Lanka, on the other hand even though has made progress in developing Mental Health Policy highlighting human rights, and dignity of people with mental illness, it has not managed to address human rights issues in their current legislation. Cultural norms are different in these countries in comparison to developed countries. The concept of ‘collectivism’ influences family involvement and decision making in these countries, therefore concerns have been raised by clinicians about some aspects of these modern mental health laws which may imply individualism, and may affect implementation in this region. However, it is worth noting that with increasing globalization, pure collectivists and individualists are possibly less a reality. On the positive side, it can be seen that the four countries included in this review are slowly addressing health and justice issues for the adequate provision of mental health care. Appropriate governance, which includes necessary policy and legislative frameworks to promote and protect the mental health of a population, can overcome barriers to effective integration of mental health care [36].

## Supplementary information

**Additional file 1.** Questionnaire.

## Data Availability

The questionnaire can be found in Additional file 1: Appendix S1.

## Abbreviations

CMHA: Central Mental Health Authority
FMHA: Federal Mental Health Authority
MHA: Mental Healthcare Act
MHL: Mental Health Legislation
MHO: Mental Health Ordinance
MHRB: Mental Health Review Board
SMHA: State Mental Health Authority
UN-CRPD: United Nations Convention on the Rights of Persons with Disabilities
WHO: World Health Organization
