# Supplementary material for: Comparing legislation for involuntary admission and treatment of mental illness in four South Asian countries
Source: Int J Ment Health Syst. 2019 Oct 24;13:67. doi: 10.1186/s13033-019-0322-7 (PMC6813093; doi:10.1186/s13033-019-0322-7)
Supplement: Supplementary file 1 — Additional file 1. Questionnaire. [file 13033_2019_322_MOESM1_ESM.docx]

**Appendix S1: Questionnaire**

Please answer yes or no and add details where appropriate.

**Criteria**

1. Does your country have a mental health act (MHA)?

Yes / No

1. What is the title of the legislation and which year was the MHA first enacted?

Title:

Year:

1. How can your MHA be accessed (e.g., via a website or a copy by email attachment)?
2. Does your country have any relevant mental health policy/ies regarding compulsory psychiatric treatment?

Yes / No

1. How can your policy be accessed (e.g., via a website or a copy by email attachment)?
2. For involuntary admission under the legislation, are there specified criteria?

Yes / No

1. If yes, what are the qualifying criteria for compulsory treatment under the legislation? (e.g., the presence of mental disorder characterized by……, risk to them or others, poor self-care)? Please detail here in point format.
2. If the legislation in your country has a criterion ‘presence of mental disorder’, please detail here the definition of ‘mental disorder’ (e.g., is it based on phenomena rather than diagnosis?). If there is no such criterion, please skip this question.
3. Are all the key terms in the legislation clearly defined?

Yes / No

1. If no, what key terms need greater clarification (e.g., any term apart from ‘mental disorder’ mentioned in the legislation that needs defining)?
2. Are some conditions or characteristics explicitly excluded in the legislation (e.g., intellectual disability, personality disorder, sexual orientation, substance abuse, political affiliation)?

Yes / No

1. If there are exclusions, what are they?
2. Does the legislation require that treatment is available for people who are detained?

Yes / No

1. If yes, what is the definition of treatment?
2. Is there any mention of the term(s) ‘capacity’/‘competency’ in the legislation? If there is, please detail here.

**Process**

1. Who may initiate compulsory treatment (e.g., a family member, member of the public, clinician, police)?
2. How many assessors (clinicians) are required to initiate compulsory treatment under the legislation? Please add details.
3. What qualifications must assessors have (if any) (e.g., medical doctor, social worker, registered psychologist, only a hospital doctor, psychiatrist)?
4. Is there provision for emergency intervention before the person is compulsory treated under the legislation?

Yes / No

1. If yes, what is the form and duration of this intervention (e.g., can be hospitalised for 72 hours)? Please provide details.
2. Are there any provision for family involvement in decision-making regarding compulsory treatment?

Yes / No

1. If yes, what are the provisions (e.g., clinicians must communicate with family members)?
2. What is the potential maximum duration of initial involuntary admission (e.g., 5 days/2 weeks etc …)?
3. Is the person entitled to automatic clinical review?

Yes / No

1. If yes, by whom and at what intervals (e.g., by any doctor or psychiatrist at 5 days, 14 days and at 3 months)?
2. Does the legislation provide independent legal review of the person’s compulsory status (e.g. tribunal court or legal review board)?
3. If yes, please explain.
4. Is the person entitled to free legal representation or advocacy (e.g., for review of compulsory treatment)?

Yes / No

1. If yes, who funds the legal representation?
2. If yes, what are the representative’s qualification (e.g., Lawyer)?
3. Does the legislation refer to human rights (e.g., incorporated international and regional human rights documents such as the Universal Declaration of Human Rights, the United Nations Convention on the Rights of Persons with Disabilities)?

Yes / No

1. If yes, please expand (e.g., international or local acts that are utilised or accessed in this respect)
2. Is there any administrative infrastructure (e.g., directors for area mental health services to ensure consistent practice in the region)?

Yes / No

1. If yes, please give details.
2. Is there any community extension of the legislation (e.g., community treatment order as an outpatient)?

Yes / No

1. If so, how long?
2. Please mention any concerns, if any, you have regarding the legislation.

Please add any extra comments regarding compulsory treatment (for example, use of physical restraints available or not, routine external monitoring by an independent body etc.)

- Name of the participant(s):
- Qualifications of the participant(s):
- Contact details:

- Would you be willing to participate as an author/moderator should the researcher publish a paper based on the survey results?

Yes / No

- Would you be willing to be identified if required either as an author/moderator:

Yes / No

- Would you like to remain anonymous?

Yes / No

- Would you like to receive feedback on the results:

Yes / No

- How would you like to be informed about the results? Please add details:
- Add extra comments or questions:

Signature:

Date:

Declaration:

None of the authors have any competing interests.
